# Concerning Operations for Otorrhœa

**Published:** 1907-10-26

**Authors:** 


### Otology.

### CONCERNING OPERATIONS FOR OTORRHOEA.

### II. STEELE'S ORIGINAL, AND THE RADICAL MASTOID, OPERATIONS.

(*Continued from page* 666.)

In previous issues we considered myringotomy and intra-meatal ossiculectomy. We now pass on to (1) Stache's original operation; (2) the radical mastoid operation, with the evolution of which, according to Politzer, the names of Schwartze, Küster, von Bergmann, Zaufal, and Stache are intimately associated.

Some misconception appears to exist as to the differences which underlie these two operations.

1. *Stache's Original Operation* is fully described in "Berlin. Klin. Wochenschr.," 1892, No. 4, and was introduced as a method of perfecting ossiculectomy by the post-aural intro-meatal route, thereby securing the incus with certainty, by extending the attic opening back into the antral cavity and effecting drainage through the deep part of the post-meatal wall. A curved incision is made from the upper end of the post-auricular groove downwards towards the apex of the mastoid. The periosteum having been divided, the soft parts are pushed forwards towards the external auditory meatus, clearly exposing the entire superior and posterior margins of the outlet of the meatal canal. The cuticular lining of the canal is detached by means of a narrow periosteal elevator and displaced forwards and downwards, displaying the tympanic membrane. Stache originally now proceeded to detach the remains of the membrane from the sulcus tympanicus, and removed the malleus. The guard which bears his name is passed from the meatus into the tympanic cavity up into the attic and back through the aditus to the antrum; the bony lamella forming the outer attic wall is removed with a gouge, while the guard protects the facial nerve from injury. The incus having been extracted, the opening in the attic is extended backwards with the guard still *in situ*, until the antrum freely opens into the meatal canal. An attempt is then made to estimate the size of the antrum by the use of various shaped probes and seekers, and, if thought necessary, the meatal opening is extended outwards, laying bare more freely the accessory cavities in the mastoid. A modification of this is to reverse the order of procedure, opening the antrum intrameatally first, before removing the outer attic wall, and extracting the ossicles. This operation has a very limited application, and was discarded by its introducer in favour of the radical method (*vide infra*). The chief objection is the inadequate exposure obtained of the antrum and accessory cavities. Moreover, it is impossible by the use of seekers alone to correctly estimate either the size of the antrum or the state of its walls. For it should be clearly understood that very considerable variations in the mastoid bone exist. We can never tell before the operation is commenced what is the size or shape of the antrum, what is its depth, exactly what relation it bears to the floor of the middle cranial fossa, or to the groove of the lateral sinus which may bulge almost to the meatal wall. Nor can we be sure of opening all the accessory cavities, in the mastoid, by the intra-meatal operation, for it is not infrequent that large cells exist in the apex, which communicate with the antrum by an almost insignificant intermediate group of cells, which latter cannot be with certainty detected by seekers alone without removal of the outer cortex of the mastoid bone.

2. *The Radical Mastoid Operation.*—In 1872 Schwartze published, with sound clinical and pathological details, his method of opening the antrum and accessory cavities of the mastoid by removal of the outer mastoid cortex. In 1889 Küster laid stress on the removal of the posterior meatal wall down to the annulus tympanicus. Soon after von Bergmann combined the Schwartze-Küster operation with Stache's tympanic and attic operation. Stache discarded his original operation and improved in detail the Schwartze-Küster-von Bergmann operation. Independently, Zaufal contributed further modifications. The result of these investigators' work is the modern radical operation.

It is convenient to consider the procedure in the following stages:—(1) The exposure of the mastoid surface; (2) the opening up of the accessory cavities in the bone; (3) the examination of the walls of the cavity; (4) the opening of the attic; (5) the removal of the ossicles and tympanic contents; (6) the examination of the labyrinthine windows, facial aqueduct, and external semi-circular canal; (7) the obliteration of the Eustachian tube; (8) the plastic operation; (9) the closure of the wound and provision of drainage; (10) the after-treatment.

*Stage* 1.—The exposure of the mastoid surface. A curved incision is made with concavity forwards and downwards, commencing at the upper end of the auricular mastoid groove, and carried backwards and downwards over the mastoid for about two inches towards the apex of the process. The temporal fascia is exposed above, but not incised owing to the troublesome bleeding from the temporal muscle which is attached to its deep surface. The periosteum is divided along the supra-meatal crest and down to the lower end of the skin incision. The temporal muscle may be pushed upwards with a periosteal elevator, together with the temporal fascia. The periosteum and soft parts of the auricle and meatal canal are detached from the anterior part of the mastoid surface, exposing the supra-meatal triangle (MacEwen). The anterior margin of the wound is held downwards and forwards by a pronged retractor. The membranous lining is detached from the roof to the postero-inferior part of the external auditory canal, but not as a rule from the anterior or antero-inferior walls. This should be done with care, lest the cuticular lining be injured, and interfere with the subsequent performance of the plastic stage of the operation.

The tympanic membrane is then displayed. To obtain a better view of the membrane and tympanic ring, Politzer advocates the use of a blunt retractor, bent to the curve of the anterior meatal wall, so that the loosened posterior wall of the membranous canal is pressed against the anterior wall.

Should a fistulous track in the skin over the mastoid exist, the incision should be made through the fistula, the walls of which should be afterwards excised. This is to be preferred to an elliptical incision enclosing the fistula.

In case the soft parts superficial to the mastoid are inflamed and œdematous, the incision should be rather more freely made.

*Stage* 2.—The opening of the accessory cavities in the bone. (After Schwartze.) The external surface of the bone is removed in layers with a gouge driven by a metal mallet. The trephine and burr have been discarded by the majority of modern surgeons. One or several pieces are removed from the supra-meatal triangle (MacEwen), working upwards and forwards, and the hollow thus formed is widened by removing the lower and posterior margin of the opening in the bone (Zaufal). The posterior meatal wall is chiselled away down to the annulus tympanicus (Küster). When the antrum is opened all bone overhanging the cavity is removed, until its walls are on a level with the superficial margin of the mastoid wound. In exposing the antrum, the middle cranial fossa and the sulcus of the ending lateral sinus may be encountered, and should be borne in mind.

*Stage* 3.—Examination of the walls of the cavity. The antrum having been freely opened from the mastoid cortex, careful survey of its walls should be made for cell cavities, all of which should be opened, even down to the apex of the mastoid, whether they contain merely mucus or swollen, inflamed, and vascular mucosa, cholesteatomatous material or pus.

To any soft or discoloured area of bone, attention should be especially directed. A fistulous track may sometimes be unexpectedly disclosed, leading from the accessory cavities to the dura. In this case the bony margin of the exposed dura should be freely removed, and the condition of the surrounding dura noted. Particular care should be taken not to interfere with the eminence of the external semi-circular canal, on the inner wall of the antrum, especially when the antrum is small. The eminence of this canal should always be recognised and its condition noted. The observation may materially affect the subsequent course of the operation.

(*To be continued.*)

